# Attenuation of circulatory shock and cerebral ischemia injury in heat stroke by combination treatment with dexamethasone and hydroxyethyl starch

**DOI:** 10.1186/2040-7378-2-19

**Published:** 2010-10-11

**Authors:** Tsai-Hsiu Yang, Mei-Fen Shih, Yi-Szu Wen, Wen-Yueh Ho, Kuen-Lin Leu, Mei-Ying Wang, Chia-Chyuan Liu

**Affiliations:** 1 Department of Health and Nutrition, Chia-Nan University of Pharmacy and Science, Tainan 71710, Taiwan; 2 Department of Pharmacy, Chia-Nan University of Pharmacy and Science, Tainan 71710, Taiwan; 3 Department of Emergency medicine, Taipei Veterans General Hospital, Taipei 11267, Taiwan; 4 Department and Institute of Cosmetic Science, Chia-Nan University of Pharmacy and Science, Tainan 71710, Taiwan

## Abstract

**Background:**

Increased systemic cytokines and elevated brain levels of monoamines, and hydroxyl radical productions are thought to aggravate the conditions of cerebral ischemia and neuronal damage during heat stroke. Dexamethasone (DXM) is a known immunosuppressive drug used in controlling inflammation, and hydroxyethyl starch (HES) is used as a volume-expanding drug in cerebral ischemia and/or cerebral injury. Acute treatment with a combined therapeutic approach has been repeatedly advocated in cerebral ischemia experiments. The aim of this study is to investigate whether the combined agent (HES and DXM) has beneficial efficacy to improve the survival time (ST) and heat stroke-induced cerebral ischemia and neuronal damage in experimental heat stroke.

**Methods:**

Urethane-anesthetized rats underwent instrumentation for the measurement of colonic temperature, mean arterial pressure (MAP), local striatal cerebral blood flow (CBF), heart rate, and neuronal damage score. The rats were exposed to an ambient temperature (43 degrees centigrade) to induce heat stroke. Concentrations of the ischemic and damage markers, dopamine, serotonin, and hydroxyl radical productions in corpus striatum, and the serum levels of interleukin-1 beta, tumor necrosis factor-alpha and malondialdehyde (MDA) were observed during heat stroke.

**Results:**

After heat stroke, the rats displayed circulatory shock (arterial hypotension), decreased CBF, increased the serum levels of cytokines and MDA, increased cerebral striatal monoamines and hydroxyl radical productions release, and severe cerebral ischemia and neuronal damage compared with those of normothermic control rats. However, immediate treatment with the combined agent at the onset of heat stroke confers significant protection against heat stroke-induced circulatory shock, systemic inflammation; cerebral ischemia, cerebral monoamines and hydroxyl radical production overload, and improves neuronal damage and the ST in rats.

**Conclusions:**

Our results suggest that the combination of a colloid substance with a volume-expanding effect and an anti-inflammatory agent may provide a better resuscitation solution for victims with heat stroke.

## Background

Unless immediately recognized and treated, heat stroke is often lethal, and victims who do survive may sustain permanent neurological damage [1]. The clinical diagnosis of heat stroke is demonstrated when hyperthermia is accompanied with circulatory shock (arterial hypotension), intracranial hypertension, and cerebral ischemia and injury [2,3]. Meanwhile, the heat stroke-induced central nervous system dysfunction includes delirium, convulsion, or coma [4]. Hence, prolonging survival time in heat stroke victims may offer more sufficient time for urgent treatment, thereby ameliorating the heat stroke-induced damage.

Several lines of evidence indicate that rodents share with humans almost the same heat stroke syndromes, such as arterial hypotension, activated inflammation, and multiorgan dysfunction (in particular, cerebral ischemia, injury, and dysfunction [5-7]. In the rodents heat stroke model, significant decrements in both mean arterial pressure (MAP) and cerebral blood flow (CBF), but increments of cerebral monoamines levels, free radical productions and systemic cytokine levels are obtained in urethane-anaesthetized rats after heat stroke [8,9]. These pathophysiological changes are known to aggravate the conditions of cerebral ischemia and neuronal damage during heat stroke in rats [10]. Activated inflammation is evidenced by overproduction of pro-inflammatory cytokines (e.g., interleukin-1*β *(IL-1*β*) and tumor necrosis factor-α (TNF-α)) in circulation of heat stroke rats [11,12]. High levels of cytokines and radicals in the peripheral blood stream, as well as excessive accumulation of glutamate, hydroxyl radicals, dopamine (DA) and serotonin (5-HT) in the central brain, correlate with the severity of circulatory shock, cerebral ischemia and neuronal damage during heat stroke in rats [6,9,13,14].

Various clinical and experimental investigations have shown that single doses of dexamethasone (DXM; exogenous glucocorticoids) or hydroxyethyl starch (HES) are extensively used in the treatment of cerebral ischemia and/or cerebral injury [15-17]. In the studies of heat stroke, systemic treatment with DXM attenuated serum IL-1*β *levels and cerebral ischemia damage, and improved survival in heat stroke [18]. Additionally, the prolongation of survival in rats with HES therapy was found to be associated with augmentation of both arterial blood pressure and CBF as well as reduction of cerebral ischemia, hypoxia, and neuronal damage during heat stroke [19]. Although, many therapeutic agents show potential promise in many animal models, the results of most single-agent clinical trials are sobering. Consequently, various authors advocate studies to estimate the efficacy of combined therapeutic approaches [20,21]. Furthermore, there is less attention to evaluate immediate effects of both DXM and HES (the combined agent) on heat stroke-induced pathophysiological changes, let alone their neuroprotective underlying influences, especially in the aspects of striatal monoamines and hydroxyl radical production release. Based on these concepts, we propose whether application of the combined agent immediate treatment has efficacy to elongate the survival time, and improve the heat stroke-induced circulatory shock, cerebral ischemia, and neuronal damage in rats. Furthermore, we also attempt to ascertain whether the neuroprotective effects of the combined agent treatment are associated with inhibition of cerebral release of glutamate, DA, 5-HT, hydroxyl radicals, and the serum IL-1*β*, TNF-α and malondialdehyde (MDA) levels during heat stroke.

## Methods

### Experimental animals

**Figure 1:**
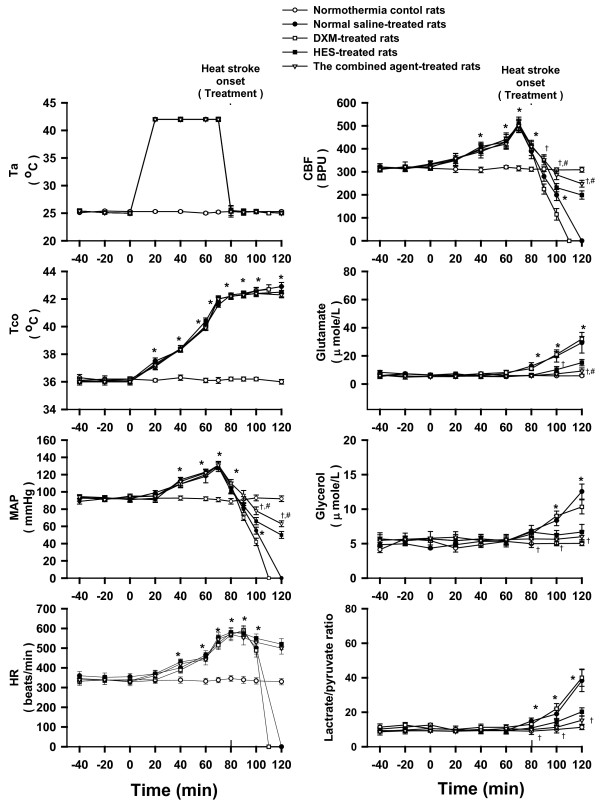
**Physiological parameters, and cellular ischemia and injury markers**. Effects of heat exposure (ambient temperature; Ta = 42°C for 80 min) on colonic temperature (Tco), mean arterial pressure (MAP), heart rate (HR), cerebral blood flow (CBF) and the extracellular concentrations of glutamate, glycerol, and lactate/pyruvate ratio of the corpus striatum in normothermic control rats (open circles), 0.9% NaCl solution (11 ml/kg)-treated (filled circles), DXM (4 mg/kg)-treated (open squares), HES (10%, 11 ml/kg)-treated (filled squares), or the combined agent (DXM+HES)-treated rats (open triangles). The dotted line indicates time of heat stroke onset and drug injection. *P <0.05, compared with normothermic control rats. ^†^*P <*0.05, compared with saline-treated rats (Ta = 42°C for 80 min).^#^*P <*0.05, compared with HES-treated rats (Ta = 42°C for 80 min) (ANOVA followed by Duncan's test).

**Figure 2:**
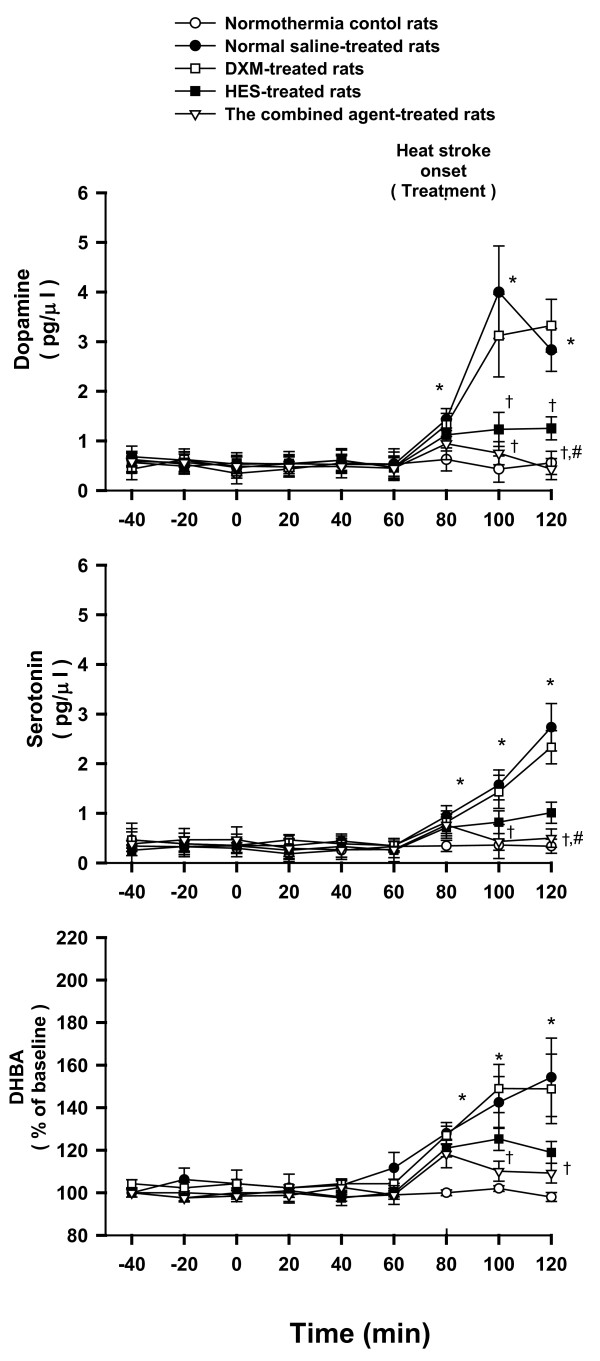
**Extracellular levels of dopamine, serotonin, and total production of DHBA**. Effects of heat exposure (ambient temperature; Ta = 42°C for 80 min) on extracellular levels of dopamine, serotonin, and total production of dihydroxybenzoic acid (DHBA) of the cerebral corpus striatum in normothermic control rats (open circles), 0.9% NaCl solution (11 ml/kg)-treated (filled circles), DXM (4 mg/kg)-treated (open squares), HES (10%, 11 ml/kg)-treated (filled squares), or the combined agent (DXM+HES)-treated rats (open triangles). The dotted line indicates time of heat stroke onset and drug injection. *P <0.05, compared with normothermic control rats. ^†^*P <*0.05, compared with saline-treated rats (Ta = 42°C for 80 min). ^#^*P <*0.05, compared with HES-treated rats (Ta = 42°C for 80 min) (ANOVA followed by Duncan's test).

**Figure 3:**
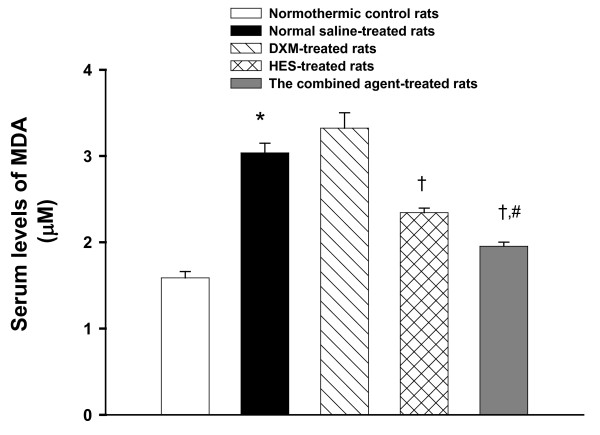
**Serum levels of MDA**. Effects of heat exposure (42°C) on serum levels of malondialdehyde (MDA) in normothermic control rats (white bar), 0.9% NaCl solution (11 ml/kg)-treated (black bar), DXM (4 mg/kg)-treated (diagonal bar), HES (10%, 11 ml/kg)-treated (cross bar) or the combined agent (DXM+HES)-treated rats (gray bar). *P <0.05, compared with normothermic control rats. ^†^*P <*0.05, compared with saline-treated rats (Ta = 42 for 80 min). ^#^*P <*0.05, compared with HES-treated rats (Ta = 42°C for 80 min) (ANOVA followed by Duncan's test). The blood samples were acquired after 100 min the initiation heat exposure in heat stroke rats or the equivalent time in normothermic controls.

**Figure 4:**
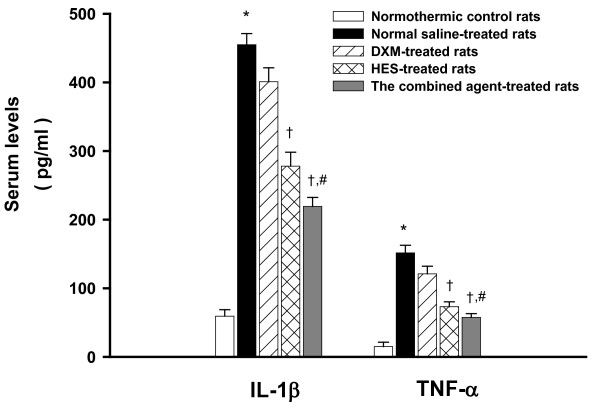
**Serum levels of IL-1β and TNF-α**. Effects of heat exposure (42°C) on serum levels of interleukin-1β (IL-1β) and tumor necrosis factor-α (TNF-α) in normothermic control rats (white bar), 0.9% NaCl solution (11 ml/kg)-treated (black bar), DXM (4 mg/kg)-treated (diagonal bar), HES (10%, 11 ml/kg)-treated (cross bar) or the combined agent (DXM+HES)-treated rats (gray bar). *P <0.05, compared with normothermic control rats. ^+^*P <*0.05, compared with saline-treated rats (Ta = 42°C for 80 min). ^#^*P <*0.05, compared with HES-treated rats (Ta = 42°C for 80 min). (ANOVA followed by Duncan's test). The blood samples were acquired after 100 min the initiation heat exposure in heat stroke rats or the equivalent time in normothermic controls.

Male *Spraque-Dawley *rats weighing between 300 and 350 g were obtained from the National Science Council of Republic of China (Taiwan). Between experiments the animals were housed individually at an ambient temperature of 24 ± 1°C with a 12-h light-dark cycle, with the lights being switched on at 0600 h. Animal chow and water were allowed *ad libitum*. All protocol were approved by the Animal Ethics Committee of the Chia-Nan University of Pharmacy and Science, Tainan, Taiwan (approbated no. CN-IACUC-94007) in accordance with the Guide for the Care and Use of Laboratory Animals of the National Institutes of Health and the guidelines of the Animal Welfare Act. Adequate anesthesia was maintained to abolish the corneal reflex and pain reflexes by tail-pinching throughout all experiments (approximately 8 hr) by a single intraperitoneal dose of urethane (1.4 g.kg^-1 ^b.w., i.p.). At the end of the experiments, control rats (and any rats that had survived heat stroke) were killed with an overdose of urethane. One hundred thirty-eight rats were used in this study. Fifty-three rats of 138 were used for examining in Table 1, Figure 1 and 2 (three premature deaths during heat stroke induction and two premature deaths during animal surgery). Forty-three rats of 138 were used for examining in Table 2 (three premature deaths during heat stroke induction). Forty-two rats of 138 were used for examining in Figure 3 and 4 (two premature deaths during heat stroke induction). No premature deaths during anesthesia.

**Table 1:** Effects of heat exposure (HE; Ta = 42°C) on both latency for the onset of heat stroke and survival time in rats treated with normal saline (NS), in rats treated with hydroxyethyl starch (HES), in rats treated with dexamethasone (DXM), and in rats treated with HES +DXM.

Treatment	Latency (mins)	Survival time (mins)
1. Rats treated with NS and kept at 24°C	> 480^†, ‡^	> 480^†, ‡, #^
2. Rats treated with NS (1 ml/kg, i.v.) and kept at 42°C	82 ± 3*	23 ± 2*^,†, ‡^
3. Rats treated with NS (11 ml/kg, i.v.) and kept at 42°C	79 ± 4*	34 ± 6*^, ‡^
4. Rats treated with HES (10%, 11 ml/kg, i.v.) and kept at 42°C	81 ± 3*	177 ± 15*^,†, #^
5. Rats treated with DXM (4 mg/kg, i.v.) and kept at 42°C	80 ± 3*	28 ± 7*^, ‡^
6. Rats treated with DXM (4 mg/kg, i.v.)+HES (10%, 11 ml/kg, i.v.) and kept at 42°C	79 ± 4*	262 ± 17*^, †, ‡, #^

NS or drugs were administered immediately after the onset of heat stroke.

Values are the means ± SEM of 8 rats per group. Groups 2-6 exposed to 42°C had heat exposure withdrawn at the onset of heat stroke.*P < 0.05, compared with the corresponding control values (rats kept at 24°C; treatment 1). (one-way ANOVA, followed by Duncan's test). ^†^P < 0.05, compared with the corresponding control values (rats treated with NS (11 ml/kg) and kept at 42°C; treatment 3). (one-way ANOVA, followed by Duncan's test). ^‡^P < 0.05, compared with the corresponding control values (rats treated with HES (11 ml/kg) and kept at 42°C; treatment 4). (one-way ANOVA, followed by Duncan's test). ^#^P < 0.05, compared with the corresponding control values (rats treated with DXM (4 mg/kg) and kept at 42°C; treatment 5). (one-way ANOVA, followed by Duncan's test).

**Table 2:** Effects of heat exposure (42°C for 80 min) plus 25 min room temperature (24°C) exposure on the neuronal damage score values of corpus striatum from normal saline-treated, dexamethasone(DXM), HES or the combined agent (DXM+HES)-treated rats^a^.

Treatment	Score of neuronal damage (0-3)
	
	striatum
1. Normal saline (1 ml/kg, i.v.)-treated rats at 24°C	0 (0, 0.75)
2. Normal saline (1 ml/kg, i.v.)-treated rats at 42°C	2 (2, 2.25)^b^
3. DXM (4 mg/kg, i.v.)-treated rats at 42°C	2 (2, 2)^b^
4. HES (10%, 11 ml/kg, i.v.)-treated rats at 42°C	1 (0.25, 1)^c^
5. DXM (4 mg/kg, i.v.)+HES (10%, 11 ml/kg, i.v.)-treated rats at 42°C	1 (0, 1)^c^

^a^Values are means ± S.E. of 8 rats per group followed by median with Q_1_and Q_3 _in parenthesis. For determination of neuronal damage score animals were killed after 80 mins heat exposure plus 25 mins room temperature exposure. The data were evaluated by a Wilcoxon signed rank test followed by Mann-Whitney test.

^b^P < 0.05, significance of difference from corresponding values (treatment 1, control rats).

^c^P < 0.05 significance of difference from corresponding values (treatment 2).

### Animal surgery and physiological parameter monitoring

Under urethane anesthesia, the right femoral artery of the rats was cannulated with polyethylene tubing (PE50) for physiological monitoring, the right femoral vein was also cannulated for blood sampling and drug administration. The animals were then positioned in a stereotaxic apparatus (Kopf model 1460) for measurement of CBF and microdialysis experiment. Physiological monitoring included colon temperature (TCO), MAP, heart rate (HR) and CBF values in the cerebral corpus striatum.

### Experimental groups

Rats were randomly assigned to one of six major groups. One group of rats (n = 8) with heat stroke received normal saline (NS) treatment (1 or 11 ml/kg body wt, 0.9% NaCl solution, i.v.) at the onset of heat stroke. Heat stroke was induced by exposing the animals to an ambient temperature of 42°C (with a relative humidity of 60% in a temperature-controlled chamber). The moment in which MAP and local CBF began to sharply decrease from their peak levels was arbitrarily defined as the onset of heat stroke, as shown in Figure 1. The interval between the start of heat exposure and onset of heat stroke were taken as values of latency. The interval between the initiation of heat stroke onset and animal death were taken as values of survival time. Another group of rats (n = 8) with heat stroke respectively received DXM 4 mg/kg i.v., HES (10%, 11 ml/kg i.v., Fresenius AG, Bad Homburg, Germany), or the combined agent [DXM (4 mg/ml/kg) together with HES (10%, 11 ml/kg)] i.v. at the onset of heat stroke. The other group of rats (n = 8) were normothermic control rats which were exposed to an ambient temperature of 24°C for at least 90 min to reach thermal equilibrium. Their physiological parameters were continuously recorded for up to 480 min (at the end of experiment). Their colon temperatures were maintained at about 36°C using the electric thermal mat before the start of experiments. The rats of these groups were continually monitored from physiological parameters (such as T_CO_, MAP, HR, and CBF) and ST during heat stroke. According to the values of ST, rats treated with the combined agent displayed the best beneficial effect on prolongation of ST in Table 1. Consequently, investigation of the heat stroke-induced circulatory shock, cerebrovascular dysfunction, cerebral ischemia and neuronal damage would be emphasized by the influence of the combined agent administration.

### Measurements of Cellular Ischemia and Injury Markers

After cannulation of vessels, the animal's head was mounted on a stereotaxic apparatus (Davis Kopf Instruments) with the nose bar positioned 3.3 mm below the horizontal line. Following a midline incision, the skull was exposed and a burr hole was made in the skull for the insertion of a dialysis probe (4 mm in length, CMA/12, Carnegie Medicine, Stockholm, Sweden). The microdialysis probe was stereotaxically implanted into the corpus striatum according to the atlas and coordinates of Paxinos and the coordinates of Paxinos and Watson (1982) [22]. As the methods described previously [19,23], an equilibrium period of 2 hours without sampling was allowed after probe implantation. The dialysis probe was perfused with Ringer's solution (147 mM Na^+^, 2.2 mM Ca^2+^, 4 mM K^+^, pH 7.0) at 2 μl/min using a CMA/100 microinfusion pump. Dialysates were collected every 20 min in a CMA140 fraction collector. Aliquots of dialysates (5 μl) were injected onto a CMA600 Microdialysis Analyzer (Carnegie Medicine) for measurement of lactate, glycerol, pyruvate and glutamate. Four analytes can be analyzed per sample and the result is displayed graphically within minutes. The thermal experiments were started after showing stabilization in four consecutive samples.

The lactate/pyruvate ratio is a well-known marker of cell ischemia, that is, an inadequate supply of oxygen and glucose. Glycerol is a marker of how severely cells are affected by the ongoing pathology. Glutamate is released from neurons during ischemia and initiates a pathological influx of calcium leading to cell damage. It is an indirect marker of cell damage in the brain, as described previously [19,23].

### Measurements of Extracellular DA and 5-HT release

Dialysates samples were collected at 20 min intervals and assayed by an HPLC system. Extracellular monoamine concentrations were assayed by HPLC combined with an electrochemical detection system. The HPLC system comprised a Beckman 126 pump (BeckmanInstru-ments) and a CMA-200 microautosampler (CMA/Microdialysis, Stockholm, Sweden), and a microbore reversed-phase column was filled with Inertsil ODS-2 (GSK-C18, 5-mmOD, 150 × 1.0-mmID). The performance of each microdialysis probe was calibrated by dialysis of a known amount of the standard mixture, and recovery of all analyses was then determined. Brain concentrations of DA and 5-HT were calculated by determining each peak height ratio relative to the internal standard and were also corrected by each probe performance. The internal standard 3-methoxy-tyramine and standard mixtures were prepared fresh daily. The mobile phase was prepared by adding 60 ml of acetonitrile, 0.42 g of SDS (2.2 mM), 200 g of sodium citrate (30 mM), 10 mg of EDTA (0.027 mM), and 1 ml of diethylamine in double-distilled water.

### Hydroxyl Radical Production Monitoring

The concentrations of hydroxyl radicals were measured by a modified procedure based on the hydroxylation of sodium salicylate by hydroxyl radicals, leading to production of 2,3-dihydroxybenzoic acid (2,3-DHBA) and 2,5-DHBA. These two compounds were then measured in dialysates by HPLC with electrochemical detection. A Ringer's solution (0.860 g of NaCl, 0.030 g of KCl and 0.033 g of CaCl_2 _per 100 ml) containing 2 mmol/l sodium salicylate was perfused through the microdialysis probe at a constant flow rate (1.2 μl/min) A reverse-phase C18 column [phase II; particle size, 3 μm; 100 × 3.2 mm (length×internal) diameter]; BioAnalytic Systems, West Lafayette, IN, U.S.A.] was used, and the mobile phase consisted of a mixture of 75 mmol/l monochloroacetic acid, 0.7 mmol/l disodium EDTA, 1.5 mmol/l sodium 1-octanesulphonate and 45 ml/l acetonitrile (pH 3.0). The retention times of 2,3-DHBA and 2,5-DHBA were 9.07 and 5.44 min respectively.

### Measurement of Serum MDA Levels

0.25 mL of serum was added to 25 μL of 0.2% BHT and 12.5 μL of 10 N NaOH (to adjust to pH~13) and incubated at 60°C for 30 min in a shaking water bath. To this was added 1.5 mL of 0.44 mol/L (or 7.2%) TCA containing 1% KI, and the mixture was placed in ice for 10 min and centrifuged (1,000*g*, 10 min). To 1 mL of the supernatant was added 0.5 mL of 0.6% TBA, and the mixture was heated at 95°C for 30 min. After cooling the mixture was extracted with 1.5 mL of *n*-butanol, and 20 μL of the butanol layer was injected to a C-18 (4.6 × 150 mm) column fitted with a guard and eluted at 1 mL/min by using 65% (v/v) 50 mM KH_2_PO_4_-KOH and 35% (v/v) methanol with spectrophotometric (532 nm) detector.

### Measurement of Serum IL-1β and TNF-α Levels

The blood samples were acquired 100 min after the initiation of heat exposure (or 20 min after the onset of heat stroke) in heat stroke rats or the equivalent time in normothermic controls. 5 ml of blood was withdrawn from the femoral vein of each rat for measurement of serum IL-1*β *or TNF-α. Blood samples were allowed to clot for 2 hours at room temperature or overnight at 2-8°C before centrifuging for 20 minutes at approximately 2000 ×g. Serum was quickly removed from these plasma samples and assayed for IL-1*β *or TNF-α immediately. The DuoSet Enzyme-linked Immunosorbent Assay (ELISA) Development System rat IL-1*β *or TNF-α kit (R&D Systems, Minneapolis, MN, USA) was used for measuring the levels of active rat IL-1*β *or TNF-α present in serum. This assay employs the quantitative colorimetric sandwich ELISA technique.

### Neuronal Damage Score

At the end of each experiment, the brain was removed, fixed in 10% neutral buffered formalin and embedded in paraffin blocks. Serial (10 μm) sections through the striatum were stained with hemotoxylin and eosin for microscopic evaluation. The extent of striatal neuronal damage was scored on a scale of 0-3, modified from the grading system of Pulsinelli et al. (1982) [24], in which 0 is normal, 1 means that ~30% of the neurons are damaged, 2 means that ~60% of that neurons are damaged, and 3 means that 100% of that neurons are damaged. Each hemisphere was evaluated independently without the examiner knowing the experimental conditions. When examined for neuronal damage in gray matter, only areas other than those invaded by probes were assessed.

### Statistical analysis

Data are presented as the mean ± SEM. Repeated-measures analysis of variance was used for factorial experiments, whereas Duncan's multiple-range test was used for post hoc multiple comparisons among means. For scoring neuronal damage, the Wilcoxon signed rank test was used when only two groups were compared. The Wilcoxon tests which convert the scores or values of a variable to ranks, require calculation of a sum of the ranks and provide critical values for the sum necessary to test the null hypothesis at a given significant level. The data were given by "median", and first and third quartile. A *P *value less than 0.05 was considered as statistical significance.

## Results

### The combined agent (DXM+HES) improves survival time in heat stroke

Table 1 summarizes the effects of heat exposure (42°C Ta for 80 min) on survival time in rat heat stroke. In anesthetized rats treated with intravenous (i.v.) doses of normal saline (NS) or drugs immediately after the onset of heat stroke, although showing no change in latency, displayed increases in survival time in some groups. For example, rats treated with an i.v. dose of 1 ml/kg or 11 ml/kg of NS had survival time values of 23 ± 2 min or 34 ± 6 min, respectively. Rats treated with HES solution at an i.v. dose of 11 ml/kg immediately after the onset of heat stroke had a survival time value of 177 ± 15 min. This increase in survival would indicate that the HES, but not the NS groups, are adequately resuscitated. Additionally, although immediate treatment with DXM (4 mg/kg) alone had no apparent beneficial effect, the combined agent (combined administration of DXM (4 mg/kg) plus HES (11 mg/kg)) immediately after the onset of heat stroke did prolong the survival time as compared with the controls (as shown in Table 1).

### The combined agent (DXM+HES) attenuates hypotension, cerebrovascular dysfunction and neuronal damage during heat stroke

The effects of heat exposure (42°C for 80 min) on several physiological parameters in NS-, DXM-, HES- and the combined agent-treated rats are shown in Figure 1. In NS-treated (11 ml/kg) and DXM (4 mg/kg)-treated groups, the values of MAP and CBF were significantly decreased at 10 or 20 min after the onset of heat stroke (or 90 or 100 min after the start of heat stress) compared with those of normothermic controls. On the other hand, the values of extracellular concentrations of glutamate, glycerol and lactate/pyruvate ratio in the corpus striatum were significantly greater than those of the normothermic controls. In HES (11 ml/kg)-treated group, rats displayed greater MAP and CBF, but lower striatal levels of glutamate, glycerol, and lactate/pyruvate ratio after onset of heat stroke than those of the NS-or DXM- treated rats. However, heat stroke-induced arterial hypotension, cerebral ischemia and increased levels of glutamate, glycerol, and lactate/pyruvate ratio in the extracellular levels of striatum were all significantly diminished by treatment with the combined agent immediately at the onset of heat stroke or 80 min after start of heat stress.

**Figure 5:**
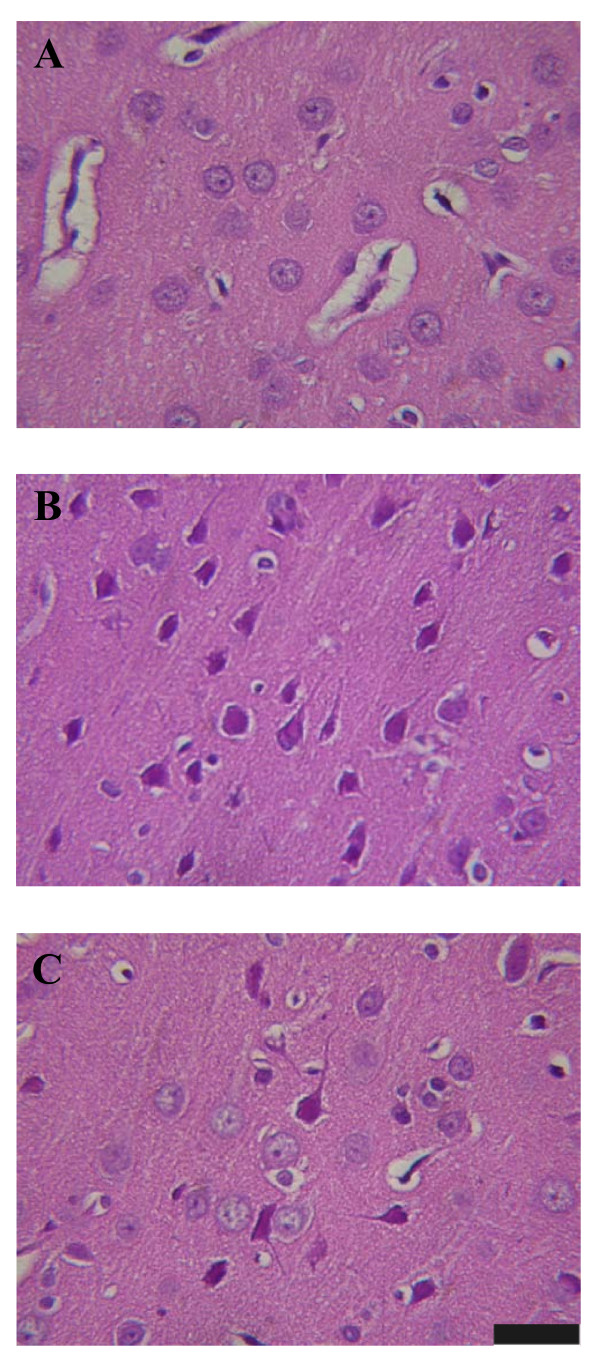
**Histological examination of neuronal damage**. The photomicrographs of the cerebral corpus striatum in a normothermic control rat treated with 0.9% NaCl solution (11 ml/kg) (A), a heat stroke rat treated with 0.9% NaCl solution (11 ml/kg) (B), or a heat stroke rat treated with the combined agent (DXM+HES) (C) immediately after the initiation of heat stroke. The striatal photomicrograph in a heat stroke rat treated with DXM (4 mg/kg) is similar to (B), and in a heat stroke rat treated with HES (10%, 11 ml/kg) is similar to (C) (data not shown). Twenty-five minutes after 80-min heat exposure, the corpus striatum of the rat treated with 0.9% NaCl solution showed cell shrinkage, pyknosis of the nucleus, and disappearance of nucleolus. After acute treatment with the combined agent, neuronal damage was reduced, as shown in C. The rats were sacrificed at 25 min after the termination of heat exposure or the equivalent time for the normothermic controls. Scale bar, 50 μm.

In separate experiments, 25 min after the onset of heat stroke, rats were sacrificed for determination of neuronal damage score in the corpus striatum. The data are summarized in Table 2. After the onset of heat stroke, rats treated with NS (11 ml/kg) displayed higher values of striatal neuronal damage [2 (2, 2.25)] compared with those of normothermic controls [0 (0, 0.75)] (as shown in Table 2 and Figure 5B). Values of striatal neuronal damage score in the DXM-treated rats and in the HES-treated rats were respectively [2 (2, 2)] and [1 (0.25, 1)]. However, with the combined agent [DXM (4 mg/kg)+HES (11 ml/kg)] acute treatment, neuroprotection ensured [1 (0, 1)]. Figure 5 shows that heat stroke-induced cell shrinkage, pyknosis of nucleus, and disappearance of nucleolus in the corpus striatum were attenuated by the combined agent (Figure 5C).

### The combined agent (DXM+HES) reduces cerebral striatal levels of dopamine, serotonin and DHBA during heat stroke

As shown in Figure 2, twenty minutes after the termination of heat stress in the NS-treated group or DXM-treated group, all the dopamine, serotonin, and DHBA (dihydroxybenzoic acid, indirectly stood for production of hydroxyl radicals) values in cerebral striatum were significantly greater than those of the normothermic controls (P < 0.05). Immediate treatment with the combined agent at the onset of heat stroke (80 min after the start of heat stress) significantly attenuated the heat stress-induced increases levels of dopamine, serotonin, and production of hydroxyl radicals in the corpus striatum.

### The combined agent (DXM+HES) reduces the levels of MDA, IL-1β and TNF-α in peripheral blood stream during heat stroke

The serum IL-1β and TNF-α, and MDA levels for normothermic controls, NS-, DXM-, HES-treated heat stroke rats, and the combined agent-treated heat stroke rats are summarized in Figure 3 and 4. It can be seen from the figures that the serum IL-1β and TNF-α, and MDA levels in NS-treated heat stroke rats were all significantly higher at 20 min after the onset of heat stroke than those in the normothermic controls. The immediate treatment with HES alone and the combined agent at the onset of heat stroke attenuated the heat stroke-induced increased serum lipid peroxidation, as well as attenuating it increased the serum levels of IL-1β and TNF-α. However, these serum levels were more significantly diminished by treatment with the combined agent immediately at the onset of heat stroke (as shown in Figure 3 and 4).

## Discussion

It has been reported that pretreatment with DXM (4 mg/kg) single dose, but not immediate treatment with DXM, before heat stress could increase the ST in rats by attenuating serum levels of interleukins [18]; however, there are fewer studies showing the immediate treatment with the combined agent at the onset of heat stroke. It will be more meaningful the combined treatment shows neuroprotection after heat stroke attacks. Although the previous results [19] have shown an insignificant therapeutic effect of DXM (4 mg/kg) administered immediately after the onset of heat stroke alone, the combination of DXM and HES provides a better therapeutic effect for rats with heat stroke in the present study. Additionally, the volume-expanding effect of the HES would be thought to improve survival during heat stroke in resuscitation. The HES would be 1.5-2 times the volume administered by 1-2 hour after it is given. In fact, in our previous study, intravenous infusion of 2-11 ml/kg of HES solution improved survival in a dose-dependent manner during heat stroke, and suggesting treatment with 11mg/kg of HES had better prolongation in survival. Hence, the HES that acts to expand the circulatory volume and a potent inflammatory agent (such as DXM) might be combined to develop an improved fluid therapy for attenuation or prevention of heat stroke-induced damage. Although our previous results indicated that the combination of HES and DXM did provide a better survival effect for rats with heat stroke, the hemodynamic, histological and biological changes by the combined treatment immediately after the onset of heat stroke were not observed in detail. In this study, administration of the combined agent indeed appears more effective to prolong the ST in rats with heat stroke, by comparison to treatment of DXM or HES alone (shown in table 1). Similarly, in agreement with the present results, treatment of the combined agent (DXM and HES) can also offer beneficial amelioration from ischemic condition and therapeutic influence in ischemic experiments [25,26]. There is evidence that cerebral ischemia (due to arterial hypotension and intracranial hypertension) may be one of the major causes to induce further damage after heat stroke onset [13,18,19]. After heat stroke induction, the CBF instantly drops from highest peak, and it is concomitant with significant increments of cerebral ischemia and injury indexes, as shown in figure 1. The lactate/pyruvate ratio is a well known marker of cellular ischemia, whereas glycerol is a marker of how severely cells are affected by ongoing pathology [27]. Excessive accumulation of glutamate has been shown in ischemic brain tissue [27]. Indeed, both present and previous results [19,23,27] have demonstrated that extracellular levels of glutamate, glycerol and lactate/pyruvate in ischemic brain are greater in heat stroke rats compared with those of normothermic controls. Meanwhile, evidences of histopathological morphology and neuronal damage scores also reveal severe neuronal damage (shown in figure 5, table 2) in heat stroke rats. However, as shown in the present results, all these heat stroke-induced cerebral ischemia and injury can be alleviated by acute treatment with the combined agent.

There were many evidences [8,14,28] that the increased DA, 5-HT and glutamate in the brain during the rat heat stroke were mediated in the development of neuronal damage. Cerebral DA, 5-HT or/and glutamate overload resulting from arterial hypotension and intracranial hypertension might be responsible for occurrence of central nervous system syndromes associated with heat stroke [14,28]. Systemic administration of dopaminergic or serotoninergic nerve depletors or receptor antagonists, or glutamate receptor antagonists cloud protect against ischemic neuronal injury in experimental heat stroke [14,28,29]. In addition, recent studies showed that the excessive accumulation of cytotoxic free radicals in the brain and oxidative stress occurred during heat stroke [9,10,30]. Evidence had accumulated to suggest that heat stroke-induced cerebral ischemia and neuronal damage might be associated with an increased production of free radicals, specifically hydroxyl radicals [9,10]. Pretreatment with hydroxyl radical scavengers, such as α-tocopherol, prevented production of hydroxyl radicals, reduced lipid peroxidation and ischemic neuronal damage in the brain of rats exposed to heat stroke and prolonged subsequent survival [31]. In brief, as demonstrated by Chang et al, after the onset of heat stroke, cessation or reduction of blood flow to the brain induced neuronal damage. This neurotoxic cascade involved overproduction of glutamate, DA, and 5-HT as well as oxidative stress in the brain [6]. Likewise, in the present study, heat stroke also produces similar increases in cerebral striatal DA, 5-HT, glutamate and hydroxyl radical production in heat stroke rats. Additionally, the heat stroke rats also displayed increased levels of lipid peroxidation in the peripheral blood stream. Indeed, according to our present findings, the heat stroke-induced high levels of DA, 5-HT, glutamate, and hydroxyl radicals in rats' corpus striatum, and the elevated plasma MDA levels can be prevented by acute treatment with the combined agent. This probably implies that the immediate administration of the combined agent during heat stroke may be mediated with the decrements of cerebral monoamines and oxidative stress to prolong the ST and improve the cerebral neuronal damage in rats.

The serum concentrations of inflammatory cytokines (such as IL-1*β *and TNF-α) are elevated in humans and animals with heat stroke [12,18,23,32]. The levels of both TNF-α and IL-1 receptors correlate well with severity of heat stroke [32,33]. The previous studies had also shown that heat stroke induced systemic and cerebral striatal productions of IL-1*β *and TNF-α in both rats and rabbits [9,31,34,35]. Indeed, as it is shown in the present results, an increase of serum IL-1*β *and TNF-α levels is observed in heat stroke rats. The increase in the levels of these inflammatory cytokines is associated with arterial hypotension, cerebral ischemia and neuronal damage. Administration of IL-1 receptor antagonists could prevent arterial hypotension and cerebral ischemic damage, and improve survival in heat stroke. Furthermore, the present results show that treatment with the combined agent significantly attenuates the heat stroke-induced overproduction of IL-1*β *and TNF-α in the serum. Meanwhile, both arterial hypotension and cerebral ischemic damage are attenuated and survival of heat stroke rats is ameliorated following acute treatment with the combined agent. The immediate administration of this combined agent might exert its protective effects by attenuating the increased plasma level of IL-1*β *and TNF-α during heat stroke.

Our results indicated that following heat stroke, arterial hypotension, decreased cerebral blood flow, increased serum levels of IL-1*β*, TNF-α and MDA, and increased striatal dopamine, serotonin and hydroxyl radicals and increased of levels of glutamate, glycerol and lactate/pyruvate ratio developed. Although HES administration alone showed a pronounced effect, it was found that treatment with the combined agent conferred a moderate further beneficial effect to ameliorate these changes, and improve neuronal damage and survival time. Various clinical and experimental investigations of stroke and brain injury have shown that HES administration might reduce brain edema and intracranial hypertension [36-38]. It was also obtained that the values of MAP, cerebral perfusion pressure (CPP), and cerebral levels of local CBF were significantly lower during heat stroke [6,27]. The maintenance of appropriate levels of CBF might be brought about by higher CPP resulting from lower intracranial pressure (ICP; possibly due to reduction in brain edema and cerebovascualr congestion) and higher MAP during development of heat stroke [39]. This raises the possibility that HES might be a beneficial treatment for heat stroke subjects with intracranial hypertension as well as decreased cerebral perfusion. As a result of present study, we see from Figure 1, 2, 3 and 4, and Table 1 and 2 that acute treatment with HES (11 ml/kg) alone at the onset of heat stroke can alleviate the heat stroke-induced arterial hypotension, cerebral monoamines and hydroxyl radical production overload, systemic inflammation, and severe cerebral ischemia and damage. However, treatment with the combined agent (both HES and DXM) has more effective therapy than treatment with HES alone to maintain appropriate levels of MAP and CBF by attenuating the heat stroke-induced abnormal physiological and pathological changes, and results in prolongation in survival (as shown in Figure 1, 2, 3 and 4, and Table 1). Therefore, HES treatment showed partial effects on those parameters after heat stroke induction. According to our present results, it is reasonable to assume that acute treatment with both HES and DXM has a better effectiveness on reducing the heat stroke-induced damage, and augmenting ST. It is not known whether HES treatment exerts its benefit effect in heat stroke by acting through attenuation of brain edema and intracranial hypertension in present study. Of course, this needs further investigation.

## Conclusions

In the present study, the heat stroke-induced increases in arterial hypotension, cerebral ischemia and neuronal damage are associated with elevated levels of DA, 5-HT, glutamate and hydroxyl radicals in rat brain, and increased circulating IL-1*β*, TNF-α and MDA in the peripheral blood stream. The immediate systemic treatment with the combined agent (both DXM and HES), in addition to attenuating the elevating levels of IL-1*β*, TNF-α and MDA in blood stream, diminishes monoamines, glutamate, and hydroxyl radical formation, and ischemia injury in the brain, and improves ST in rats with heat stroke. Our results suggest that the combination of a colloid substance with a volume-expanding effect and an anti-inflammatory agent may provide a better resuscitation solution for victims with heat stroke.

## Abbreviations

CBF: cerebral blood flow; DA: dopamine; DHBA: dihydroxybenzoic acid; DXM: dexamethasone; ELISA: enzyme-linked immunosorbent assay; HES: hydroxyl starch; HR: heart rate; 5-HT: serotonin; IL-1*β *: interleukin-1*β*; MAP: mean arterial pressure; NS: normal saline; ST: survival time; Tco: colon temperature; TNF-α: tumor necrosis factor-α; ICP: intracranial pressure; CPP: cerebral perfusion pressure.

## Competing interests

The authors declare that they have no competing interests.

## Authors' contributions

All authors have read and approved the final manuscript. YTH and WMY operated the animals, assessed the neuron damage score and interpreted the data. HWY and LKL collected blood samples and performed the ELISA. SMF and WYS provided DXM and HES, and finalized the manuscript. YTH and LCC conceived the experiments, funded the project and wrote the manuscript.
